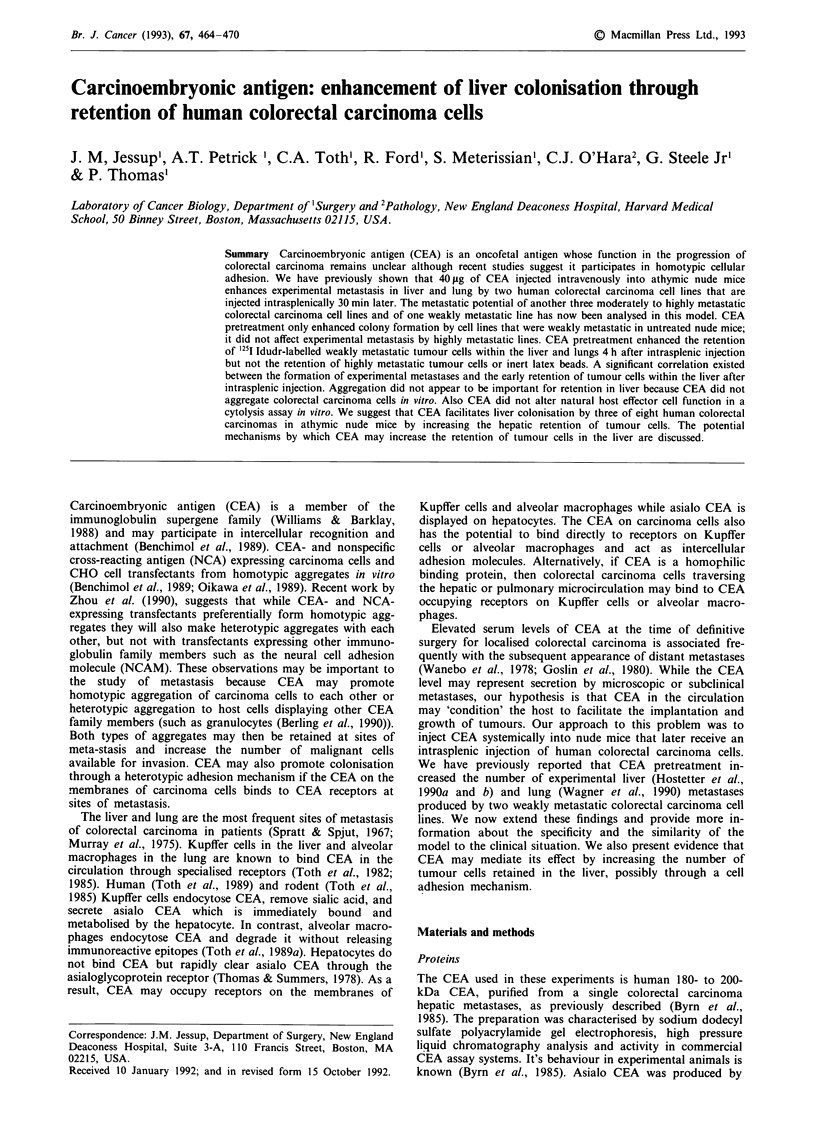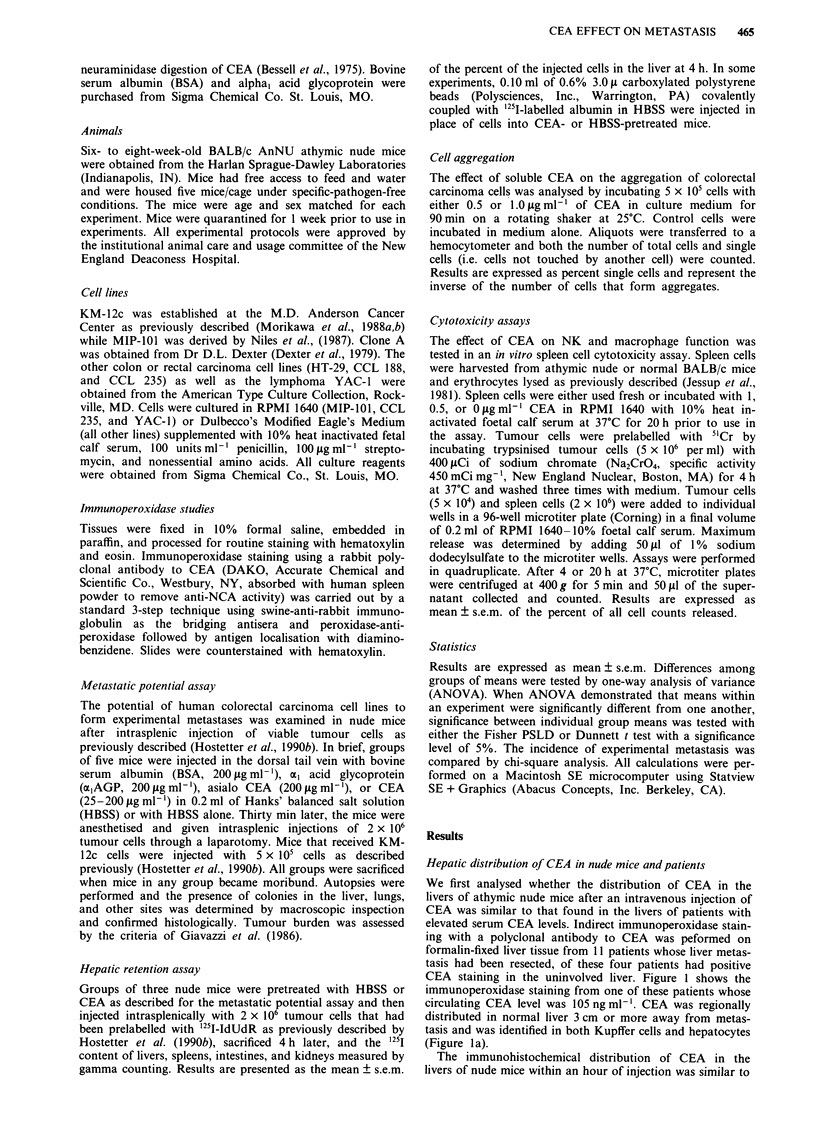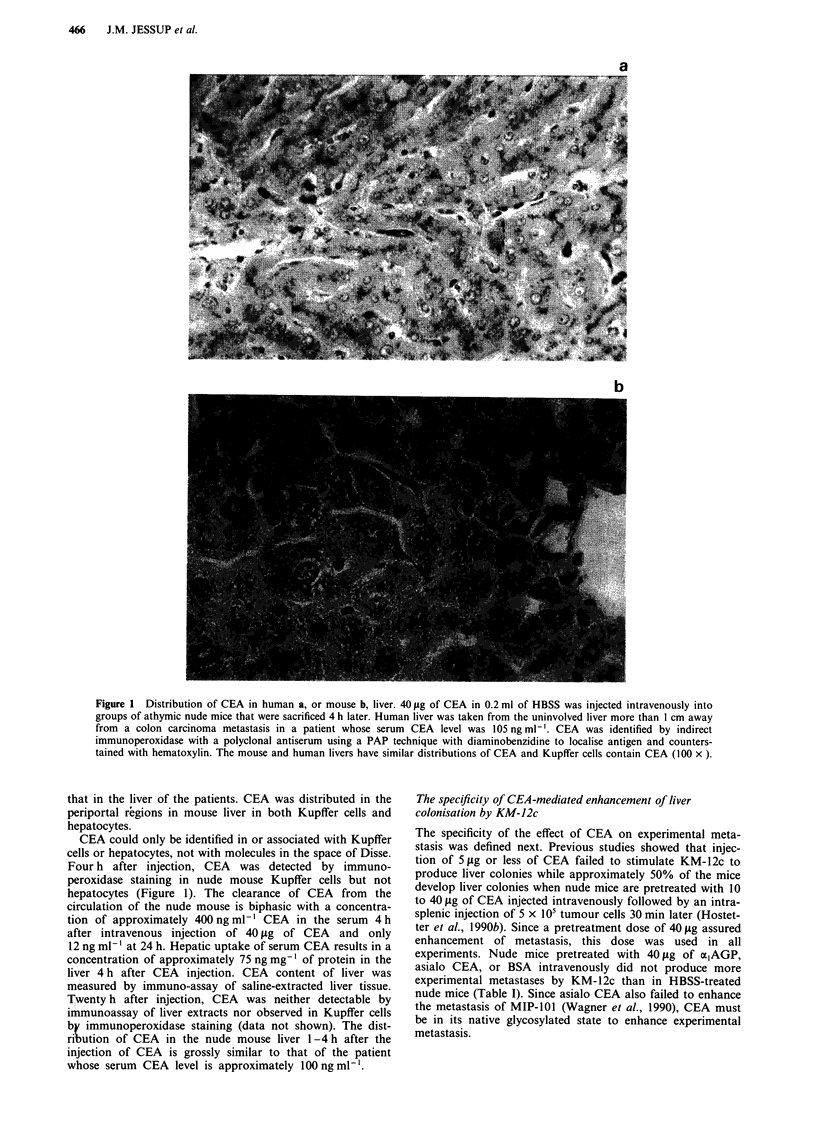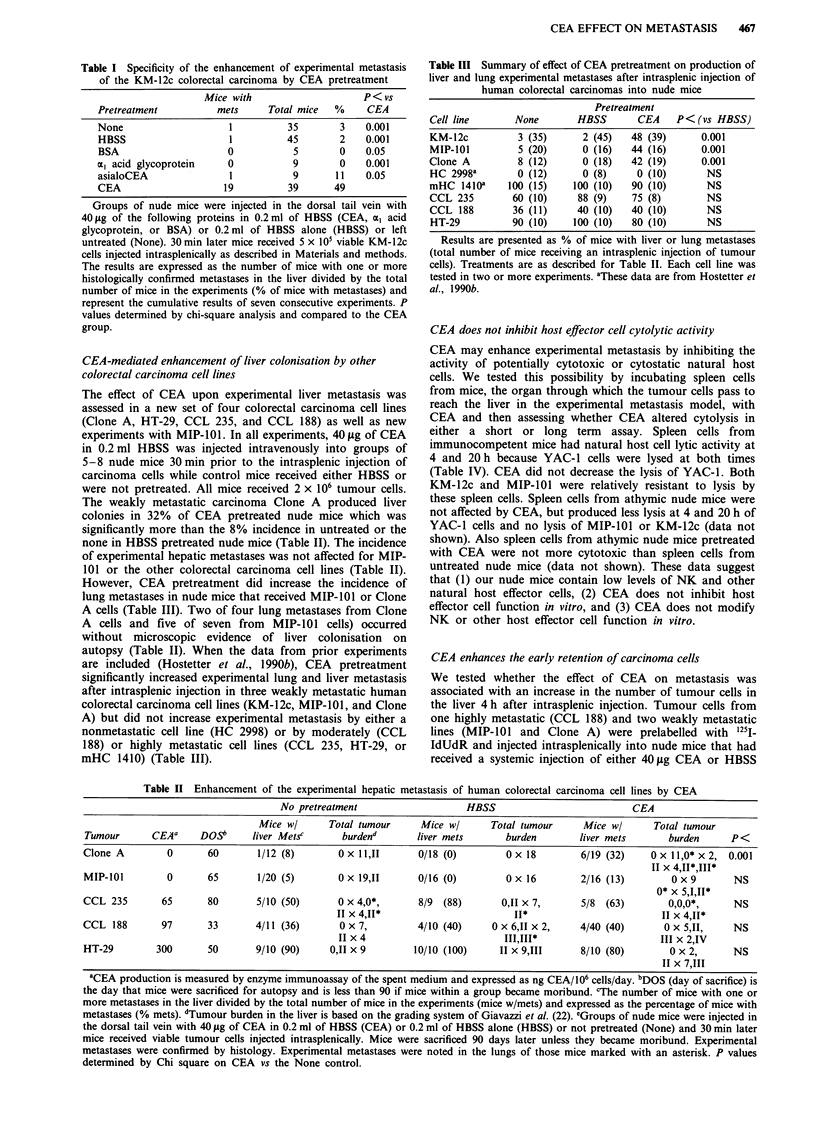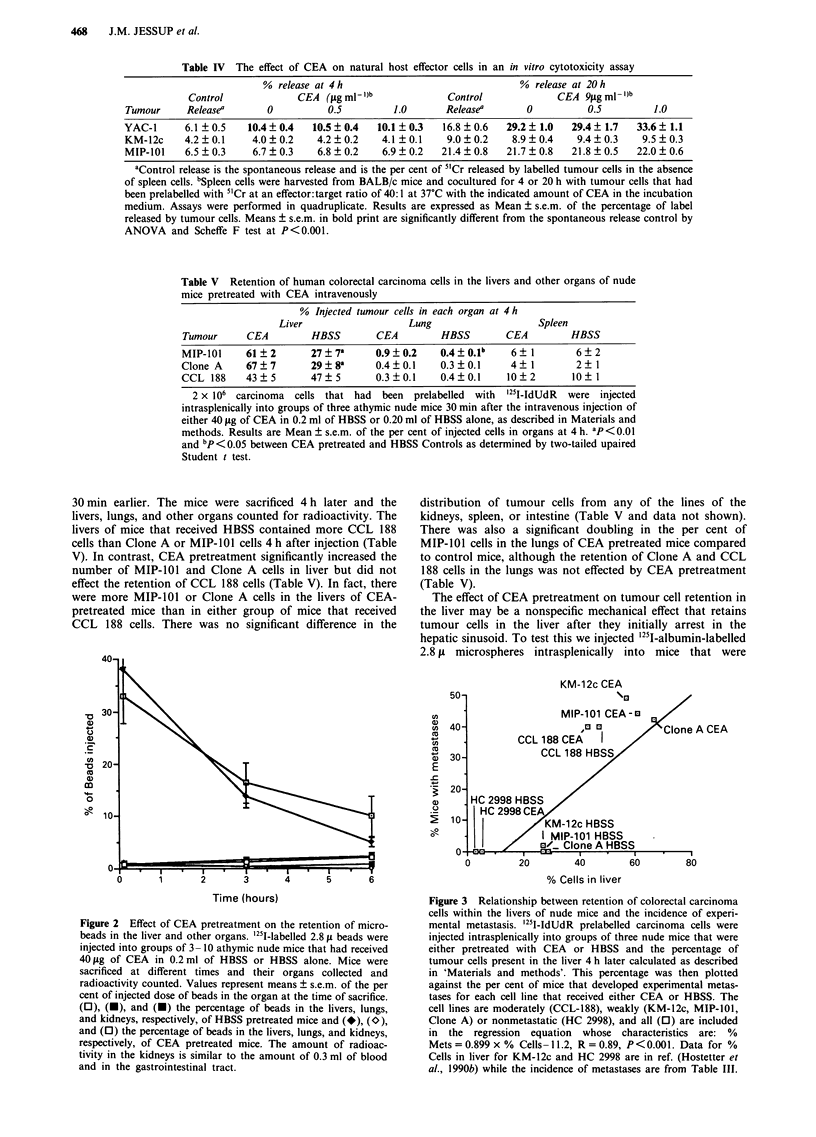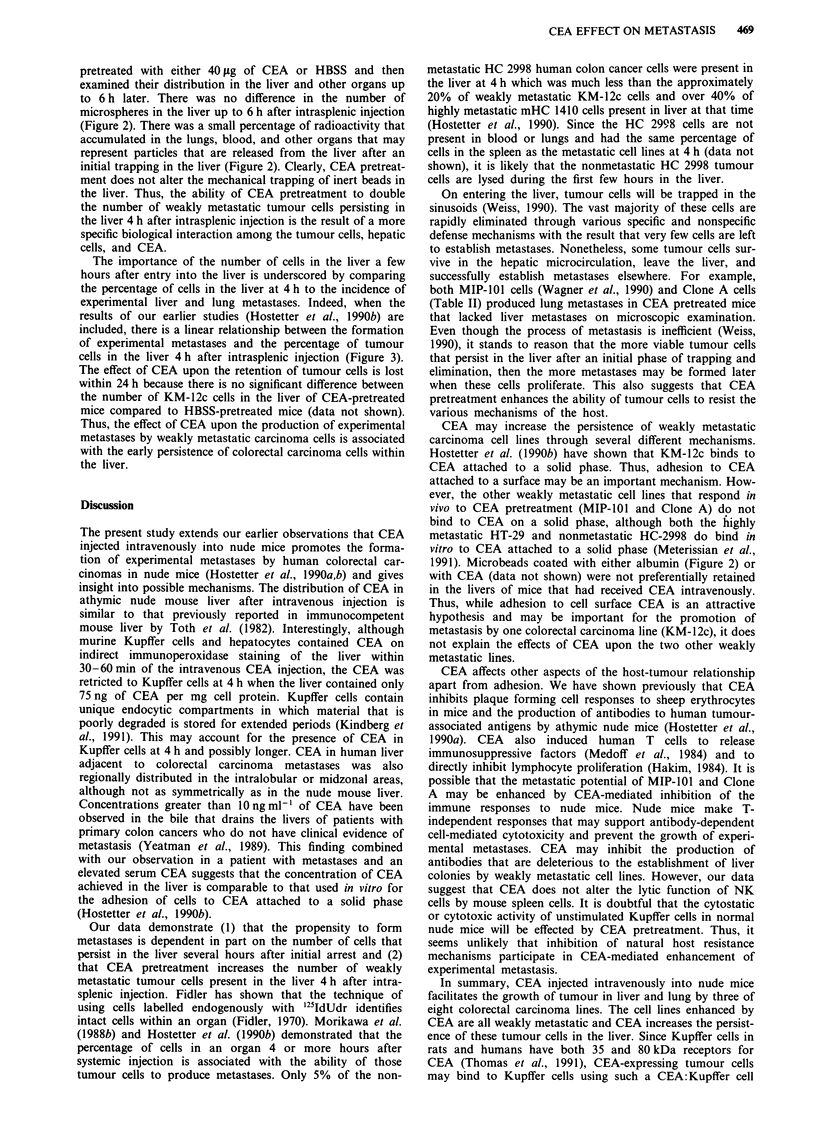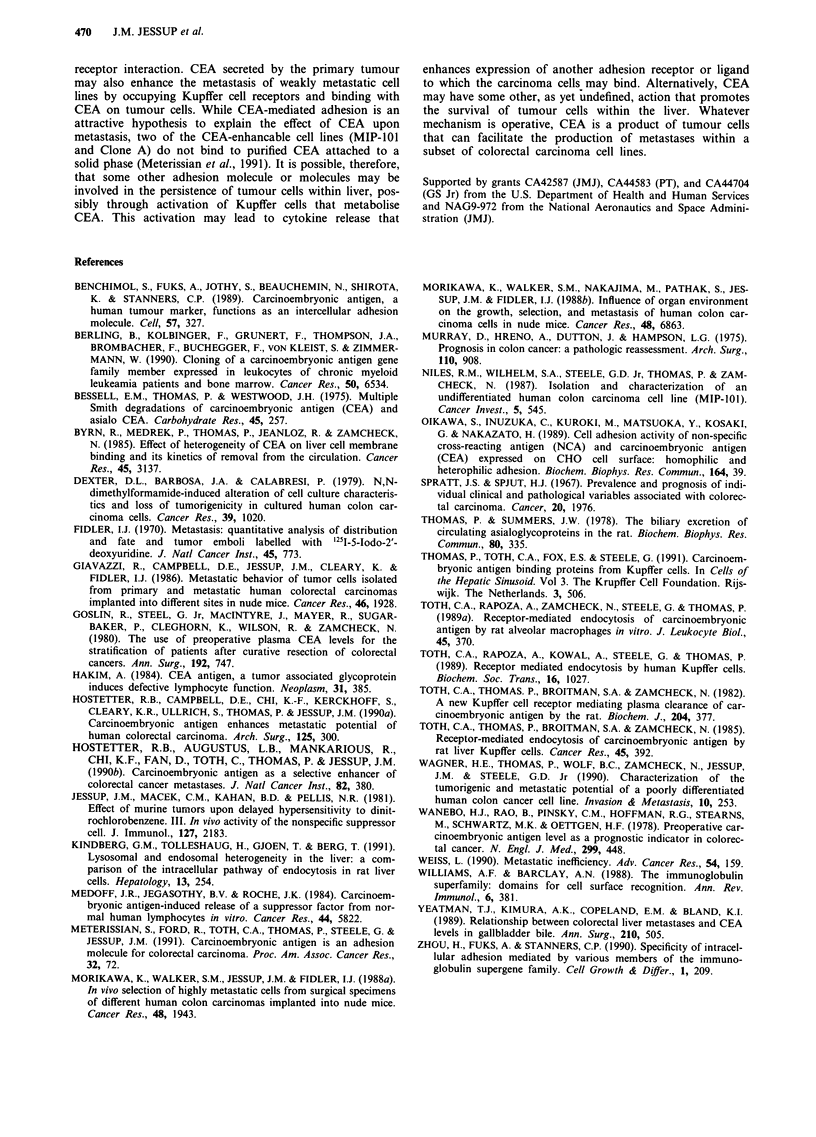# Carcinoembryonic antigen: enhancement of liver colonisation through retention of human colorectal carcinoma cells.

**DOI:** 10.1038/bjc.1993.88

**Published:** 1993-03

**Authors:** J. M. Jessup, A. T. Petrick, C. A. Toth, R. Ford, S. Meterissian, C. J. O'Hara, G. Steele, P. Thomas

**Affiliations:** Department of Surgery, New England Deaconess Hospital, Harvard Medical School, Boston, Massachusetts 02115.

## Abstract

**Images:**


Br. J. Cancer (1993), 67, 464-470 © Macmillan Press Ltd., 1993

### Carcinoembryonic antigen: enhancement of liver colonisation through retention of human colorectal carcinoma cells

J. M, Jessup[1], A.T. Petrick [1], C.A. Toth[1], R. Ford[1], S. Meterissian[1], C.J. O'Hara[2], G. Steele Jr[1] & P. Thomas[1]

*Laboratory of Cancer Biology, Department of [1]Surgery and [2]Pathology, New England Deaconess Hospital, Harvard Medical School, 50 Binney Street, Boston, Massachusetts 02115, USA.*

**Summary** Carcinoembryonic antigen (CEA) is an oncofetal antigen whose function in the progression of colorectal carcinoma remains unclear although recent studies suggest it participates in homotypic cellular adhesion. We have previously shown that 40 µg of CEA injected intravenously into athymic nude mice enhances experimental metastasis in liver and lung by two human colorectal carcinoma cell lines that are injected intrasplenically 30 min later. The metastatic potential of another three moderately to highly metastatic colorectal carcinoma cell lines and of one weakly metastatic line has now been analysed in this model. CEA pretreatment only enhanced colony formation by cell lines that were weakly metastatic in untreated nude mice; it did not affect experimental metastasis by highly metastatic lines. CEA pretreatment enhanced the retention of $^{125}$I Idudr-labelled weakly metastatic tumour cells within the liver and lungs 4 h after intrasplenic injection but not the retention of highly metastatic tumour cells or inert latex beads. A significant correlation existed between the formation of experimental metastases and the early retention of tumour cells within the liver after intrasplenic injection. Aggregation did not appear to be important for retention in liver because CEA did not aggregate colorectal carcinoma cells *in vitro*. Also CEA did not alter natural host effector cell function in a cytolysis assay *in vitro*. We suggest that CEA facilitates liver colonisation by three of eight human colorectal carcinomas in athymic nude mice by increasing the hepatic retention of tumour cells. The potential mechanisms by which CEA may increase the retention of tumour cells in the liver are discussed.

Carcinoembryonic antigen (CEA) is a member of the immunoglobulin supergene family (Williams & Barklay, 1988) and may participate in intercellular recognition and attachment (Benchimol *et al.*, 1989). CEA- and nonspecific cross-reacting antigen (NCA) expressing carcinoma cells and CHO cell transfectants from homotypic aggregates *in vitro* (Benchimol *et al.*, 1989; Oikawa *et al.*, 1989). Recent work by Zhou *et al.* (1990), suggests that while CEA- and NCA-expressing transfectants preferentially form homotypic aggregates they will also make heterotypic aggregates with each other, but not with transfectants expressing other immunoglobulin family members such as the neural cell adhesion molecule (NCAM). These observations may be important to the study of metastasis because CEA may promote homotypic aggregation of carcinoma cells to each other or heterotypic aggregation to host cells displaying other CEA family members (such as granulocytes (Berling *et al.*, 1990)). Both types of aggregates may then be retained at sites of meta-stasis and increase the number of malignant cells available for invasion. CEA may also promote colonisation through a heterotypic adhesion mechanism if the CEA on the membranes of carcinoma cells binds to CEA receptors at sites of metastasis.

The liver and lung are the most frequent sites of metastasis of colorectal carcinoma in patients (Spratt & Spjut, 1967; Murray *et al.*, 1975). Kupffer cells in the liver and alveolar macrophages in the lung are known to bind CEA in the circulation through specialised receptors (Toth *et al.*, 1982; 1985). Human (Toth *et al.*, 1989) and rodent (Toth *et al.*, 1985) Kupffer cells endocytose CEA, remove sialic acid, and secrete asialo CEA which is immediately bound and metabolised by the hepatocyte. In contrast, alveolar macrophages endocytose CEA and degrade it without releasing immunoreactive epitopes (Toth *et al.*, 1989*a*). Hepatocytes do not bind CEA but rapidly clear asialo CEA through the asialoglycoprotein receptor (Thomas & Summers, 1978). As a result, CEA may occupy receptors on the membranes of Kupffer cells and alveolar macrophages while asialo CEA is displayed on hepatocytes. The CEA on carcinoma cells also has the potential to bind directly to receptors on Kupffer cells or alveolar macrophages and act as intercellular adhesion molecules. Alternatively, if CEA is a homophilic binding protein, then colorectal carcinoma cells traversing the hepatic or pulmonary microcirculation may bind to CEA occupying receptors on Kupffer cells or alveolar macrophages.

Elevated serum levels of CEA at the time of definitive surgery for localised colorectal carcinoma is associated frequently with the subsequent appearance of distant metastases (Wanebo *et al.*, 1978; Goslin *et al.*, 1980). While the CEA level may represent secretion by microscopic or subclinical metastases, our hypothesis is that CEA in the circulation may 'condition' the host to facilitate the implantation and growth of tumours. Our approach to this problem was to inject CEA systemically into nude mice that later receive an intrasplenic injection of human colorectal carcinoma cells. We have previously reported that CEA pretreatment increased the number of experimental liver (Hostetter *et al.*, 1990*a* and *b*) and lung (Wagner *et al.*, 1990) metastases produced by two weakly metastatic colorectal carcinoma cell lines. We now extend these findings and provide more information about the specificity and the similarity of the model to the clinical situation. We also present evidence that CEA may mediate its effect by increasing the number of tumour cells retained in the liver, possibly through a cell adhesion mechanism.

### Materials and methods

### *Proteins*

The CEA used in these experiments is human 180- to 200-kDa CEA, purified from a single colorectal carcinoma hepatic metastases, as previously described (Byrn *et al.*, 1985). The preparation was characterised by sodium dodecyl sulfate polyacrylamide gel electrophoresis, high pressure liquid chromatography analysis and activity in commercial CEA assay systems. It's behaviour in experimental animals is known (Byrn *et al.*, 1985). Asialo CEA was produced by

Correspondence: J.M. Jessup, Department of Surgery, New England Deaconess Hospital, Suite 3-A, 110 Francis Street, Boston, MA 02215, USA.
Received 10 January 1992; and in revised form 15 October 1992.

neuraminidase digestion of CEA (Bessell *et al.*, 1975). Bovine serum albumin (BSA) and alpha$_1$ acid glycoprotein were purchased from Sigma Chemical Co. St. Louis, MO.

*Animals*

Six- to eight-week-old BALB/c AnNU athymic nude mice were obtained from the Harlan Sprague-Dawley Laboratories (Indianapolis, IN). Mice had free access to feed and water and were housed five mice/cage under specific-pathogen-free conditions. The mice were age and sex matched for each experiment. Mice were quarantined for 1 week prior to use in experiments. All experimental protocols were approved by the institutional animal care and usage committee of the New England Deaconess Hospital.

*Cell lines*

KM-12c was established at the M.D. Anderson Cancer Center as previously described (Morikawa *et al.*, 1988*a,b*) while MIP-101 was derived by Niles *et al.*, (1987). Clone A was obtained from Dr D.L. Dexter (Dexter *et al.*, 1979). The other colon or rectal carcinoma cell lines (HT-29, CCL 188, and CCL 235) as well as the lymphoma YAC-1 were obtained from the American Type Culture Collection, Rockville, MD. Cells were cultured in RPMI 1640 (MIP-101, CCL 235, and YAC-1) or Dulbecco's Modified Eagle's Medium (all other lines) supplemented with 10% heat inactivated fetal calf serum, 100 units ml$^{-1}$ penicillin, 100 $\mu$g ml$^{-1}$ streptomycin, and nonessential amino acids. All culture reagents were obtained from Sigma Chemical Co., St. Louis, MO.

*Immunoperoxidase studies*

Tissues were fixed in 10% formal saline, embedded in paraffin, and processed for routine staining with hematoxylin and eosin. Immunoperoxidase staining using a rabbit polyclonal antibody to CEA (DAKO, Accurate Chemical and Scientific Co., Westbury, NY, absorbed with human spleen powder to remove anti-NCA activity) was carried out by a standard 3-step technique using swine-anti-rabbit immunoglobulin as the bridging antisera and peroxidase-anti-peroxidase followed by antigen localisation with diaminobenzidene. Slides were counterstained with hematoxylin.

*Metastatic potential assay*

The potential of human colorectal carcinoma cell lines to form experimental metastases was examined in nude mice after intrasplenic injection of viable tumour cells as previously described (Hostetter *et al.*, 1990*b*). In brief, groups of five mice were injected in the dorsal tail vein with bovine serum albumin (BSA, 200 $\mu$g ml$^{-1}$), $\alpha_1$ acid glycoprotein ($\alpha_1$AGP, 200 $\mu$g ml$^{-1}$), asialo CEA (200 $\mu$g ml$^{-1}$), or CEA (25–200 $\mu$g ml$^{-1}$) in 0.2 ml of Hanks' balanced salt solution (HBSS) or with HBSS alone. Thirty min later, the mice were anesthetised and given intrasplenic injections of $2 \times 10^6$ tumour cells through a laparotomy. Mice that received KM-12c cells were injected with $5 \times 10^5$ cells as described previously (Hostetter *et al.*, 1990*b*). All groups were sacrificed when mice in any group became moribund. Autopsies were performed and the presence of colonies in the liver, lungs, and other sites was determined by macroscopic inspection and confirmed histologically. Tumour burden was assessed by the criteria of Giavazzi et *al.* (1986).

*Hepatic retention assay*

Groups of three nude mice were pretreated with HBSS or CEA as described for the metastatic potential assay and then injected intrasplenically with $2 \times 10^6$ tumour cells that had been prelabelled with $^{125}$I-IdUdR as previously described by Hostetter *et al.* (1990*b*), sacrificed 4 h later, and the $^{125}$I content of livers, spleens, intestines, and kidneys measured by gamma counting. Results are presented as the mean ± s.e.m. of the percent of the injected cells in the liver at 4 h. In some experiments, 0.10 ml of 0.6% 3.0 $\mu$ carboxylated polystyrene beads (Polysciences, Inc., Warrington, PA) covalently coupled with $^{125}$I-labelled albumin in HBSS were injected in place of cells into CEA- or HBSS-pretreated mice.

*Cell aggregation*

The effect of soluble CEA on the aggregation of colorectal carcinoma cells was analysed by incubating $5 \times 10^5$ cells with either 0.5 or 1.0 $\mu$g ml$^{-1}$ of CEA in culture medium for 90 min on a rotating shaker at 25°C. Control cells were incubated in medium alone. Aliquots were transferred to a hemocytometer and both the number of total cells and single cells (i.e. cells not touched by another cell) were counted. Results are expressed as percent single cells and represent the inverse of the number of cells that form aggregates.

*Cytotoxicity assays*

The effect of CEA on NK and macrophage function was tested in an *in vitro* spleen cell cytotoxicity assay. Spleen cells were harvested from athymic nude or normal BALB/c mice and erythrocytes lysed as previously described (Jessup *et al.*, 1981). Spleen cells were either used fresh or incubated with 1, 0.5, or 0 $\mu$g ml$^{-1}$ CEA in RPMI 1640 with 10% heat inactivated foetal calf serum at 37°C for 20 h prior to use in the assay. Tumour cells were prelabelled with $^{51}$Cr by incubating trypsinised tumour cells ($5 \times 10^6$ per ml) with 400 $\mu$Ci of sodium chromate ($Na_2CrO_4$, specific activity 450 mCi mg$^{-1}$, New England Nuclear, Boston, MA) for 4 h at 37°C and washed three times with medium. Tumour cells ($5 \times 10^4$) and spleen cells ($2 \times 10^6$) were added to individual wells in a 96-well microtiter plate (Corning) in a final volume of 0.2 ml of RPMI 1640–10% foetal calf serum. Maximum release was determined by adding 50 $\mu$l of 1% sodium dodecylsulfate to the microtiter wells. Assays were performed in quadruplicate. After 4 or 20 h at 37°C, microtiter plates were centrifuged at 400 *g* for 5 min and 50 $\mu$l of the supernatant collected and counted. Results are expressed as mean ± s.e.m. of the percent of all cell counts released.

*Statistics*

Results are expressed as mean ± s.e.m. Differences among groups of means were tested by one-way analysis of variance (ANOVA). When ANOVA demonstrated that means within an experiment were significantly different from one another, significance between individual group means was tested with either the Fisher PSLD or Dunnett *t* test with a significance level of 5%. The incidence of experimental metastasis was compared by chi-square analysis. All calculations were performed on a Macintosh SE microcomputer using Statview SE + Graphics (Abacus Concepts, Inc. Berkeley, CA).

### Results

*Hepatic distribution of CEA in nude mice and patients*

We first analysed whether the distribution of CEA in the livers of athymic nude mice after an intravenous injection of CEA was similar to that found in the livers of patients with elevated serum CEA levels. Indirect immunoperoxidase staining with a polyclonal antibody to CEA was peformed on formalin-fixed liver tissue from 11 patients whose liver metastasis had been resected, of these four patients had positive CEA staining in the uninvolved liver. Figure 1 shows the immunoperoxidase staining from one of these patients whose circulating CEA level was 105 ng ml$^{-1}$. CEA was regionally distributed in normal liver 3 cm or more away from metastasis and was identified in both Kupffer cells and hepatocytes (Figure 1a).

The immunohistochemical distribution of CEA in the livers of nude mice within an hour of injection was similar to

**Figure 1** Distribution of CEA in human **a**, or mouse **b**, liver. 40 $\mu$g of CEA in 0.2 ml of HBSS was injected intravenously into groups of athymic nude mice that were sacrificed 4 h later. Human liver was taken from the uninvolved liver more than 1 cm away from a colon carcinoma metastasis in a patient whose serum CEA level was 105 ng ml$^{-1}$. CEA was identified by indirect immunoperoxidase with a polyclonal antiserum using a PAP technique with diaminobenzidine to localise antigen and counterstained with hematoxylin. The mouse and human livers have similar distributions of CEA and Kupffer cells contain CEA (100 ×).

that in the liver of the patients. CEA was distributed in the periportal regions in mouse liver in both Kupffer cells and hepatocytes.

CEA could only be identified in or associated with Kupffer cells or hepatocytes, not with molecules in the space of Disse. Four h after injection, CEA was detected by immunoperoxidase staining in nude mouse Kupffer cells but not hepatocytes (Figure 1). The clearance of CEA from the circulation of the nude mouse is biphasic with a concentration of approximately 400 ng ml$^{-1}$ CEA in the serum 4 h after intravenous injection of 40 $\mu$g of CEA and only 12 ng ml$^{-1}$ at 24 h. Hepatic uptake of serum CEA results in a concentration of approximately 75 ng mg$^{-1}$ of protein in the liver 4 h after CEA injection. CEA content of liver was measured by immuno-assay of saline-extracted liver tissue. Twenty h after injection, CEA was neither detectable by immunoassay of liver extracts nor observed in Kupffer cells by immunoperoxidase staining (data not shown). The distribution of CEA in the nude mouse liver 1–4 h after the injection of CEA is grossly similar to that of the patient whose serum CEA level is approximately 100 ng ml$^{-1}$.

### *The specificity of CEA-mediated enhancement of liver colonisation by KM-12c*

The specificity of the effect of CEA on experimental metastasis was defined next. Previous studies showed that injection of 5 $\mu$g or less of CEA failed to stimulate KM-12c to produce liver colonies while approximately 50% of the mice develop liver colonies when nude mice are pretreated with 10 to 40 $\mu$g of CEA injected intravenously followed by an intrasplenic injection of 5 × 10$^5$ tumour cells 30 min later (Hostetter *et al.*, 1990*b*). Since a pretreatment dose of 40 $\mu$g assured enhancement of metastasis, this dose was used in all experiments. Nude mice pretreated with 40 $\mu$g of $\alpha_1$AGP, asialo CEA, or BSA intravenously did not produce more experimental metastases by KM-12c than in HBSS-treated nude mice (Table I). Since asialo CEA also failed to enhance the metastasis of MIP-101 (Wagner *et al.*, 1990), CEA must be in its native glycosylated state to enhance experimental metastasis.

**Table I** Specificity of the enhancement of experimental metastasis of the KM-12c colorectal carcinoma by CEA pretreatment

| *Pretreatment* | *Mice with mets* | *Total mice* | *%* | *P< vs CEA* |
|---|---|---|---|---|
| None | 1 | 35 | 3 | 0.001 |
| HBSS | 1 | 45 | 2 | 0.001 |
| BSA | 0 | 5 | 0 | 0.05 |
| $\alpha_1$ acid glycoprotein | 0 | 9 | 0 | 0.001 |
| asialoCEA | 1 | 9 | 11 | 0.05 |
| CEA | 19 | 39 | 49 | |

Groups of nude mice were injected in the dorsal tail vein with 40 μg of the following proteins in 0.2 ml of HBSS (CEA, $\alpha_1$ acid glycoprotein, or BSA) or 0.2 ml of HBSS alone (HBSS) or left untreated (None). 30 min later mice received $5 \times 10^5$ viable KM-12c cells injected intrasplenically as described in Materials and methods. The results are expressed as the number of mice with one or more histologically confirmed metastases in the liver divided by the total number of mice in the experiments (% of mice with metastases) and represent the cumulative results of seven consecutive experiments. *P* values determined by chi-square analysis and compared to the CEA group.

### *CEA-mediated enhancement of liver colonisation by other colorectal carcinoma cell lines*

The effect of CEA upon experimental liver metastasis was assessed in a new set of four colorectal carcinoma cell lines (Clone A, HT-29, CCL 235, and CCL 188) as well as new experiments with MIP-101. In all experiments, 40 μg of CEA in 0.2 ml HBSS was injected intravenously into groups of 5–8 nude mice 30 min prior to the intrasplenic injection of carcinoma cells while control mice received either HBSS or were not pretreated. All mice received $2 \times 10^6$ tumour cells. The weakly metastatic carcinoma Clone A produced liver colonies in 32% of CEA pretreated nude mice which was significantly more than the 8% incidence in untreated or the none in HBSS pretreated nude mice (Table II). The incidence of experimental hepatic metastases was not affected for MIP-101 or the other colorectal carcinoma cell lines (Table II). However, CEA pretreatment did increase the incidence of lung metastases in nude mice that received MIP-101 or Clone A cells (Table III). Two of four lung metastases from Clone A cells and five of seven from MIP-101 cells) occurred without microscopic evidence of liver colonisation on autopsy (Table II). When the data from prior experiments are included (Hostetter *et al.*, 1990*b*), CEA pretreatment significantly increased experimental lung and liver metastasis after intrasplenic injection in three weakly metastatic human colorectal carcinoma cell lines (KM-12c, MIP-101, and Clone A) but did not increase experimental metastasis by either a nonmetastatic cell line (HC 2998) or by moderately (CCL 188) or highly metastatic cell lines (CCL 235, HT-29, or mHC 1410) (Table III).

**Table III** Summary of effect of CEA pretreatment on production of liver and lung experimental metastases after intrasplenic injection of human colorectal carcinomas into nude mice

| | *Pretreatment* | | | |
|---|---|---|---|---|
| *Cell line* | *None* | *HBSS* | *CEA* | *P< (vs HBSS)* |
| KM-12c | 3 (35) | 2 (45) | 48 (39) | 0.001 |
| MIP-101 | 5 (20) | 0 (16) | 44 (16) | 0.001 |
| Clone A | 8 (12) | 0 (18) | 42 (19) | 0.001 |
| HC 2998[a] | 0 (12) | 0 (8) | 0 (10) | NS |
| mHC 1410[a] | 100 (15) | 100 (10) | 90 (10) | NS |
| CCL 235 | 60 (10) | 88 (9) | 75 (8) | NS |
| CCL 188 | 36 (11) | 40 (10) | 40 (10) | NS |
| HT-29 | 90 (10) | 100 (10) | 80 (10) | NS |

Results are presented as % of mice with liver or lung metastases (total number of mice receiving an intrasplenic injection of tumour cells). Treatments are as described for Table II. Each cell line was tested in two or more experiments. [a]These data are from Hostetter *et al.*, 1990*b*.

### *CEA does not inhibit host effector cell cytolytic activity*

CEA may enhance experimental metastasis by inhibiting the activity of potentially cytotoxic or cytostatic natural host cells. We tested this possibility by incubating spleen cells from mice, the organ through which the tumour cells pass to reach the liver in the experimental metastasis model, with CEA and then assessing whether CEA altered cytolysis in either a short or long term assay. Spleen cells from immunocompetent mice had natural host cell lytic activity at 4 and 20 h because YAC-1 cells were lysed at both times (Table IV). CEA did not decrease the lysis of YAC-1. Both KM-12c and MIP-101 were relatively resistant to lysis by these spleen cells. Spleen cells from athymic nude mice were not affected by CEA, but produced less lysis at 4 and 20 h of YAC-1 cells and no lysis of MIP-101 or KM-12c (data not shown). Also spleen cells from athymic nude mice pretreated with CEA were not more cytotoxic than spleen cells from untreated nude mice (data not shown). These data suggest that (1) our nude mice contain low levels of NK and other natural host effector cells, (2) CEA does not inhibit host effector cell function *in vitro*, and (3) CEA does not modify NK or other host effector cell function *in vitro*.

### *CEA enhances the early retention of carcinoma cells*

We tested whether the effect of CEA on metastasis was associated with an increase in the number of tumour cells in the liver 4 h after intrasplenic injection. Tumour cells from one highly metastatic (CCL 188) and two weakly metastatic lines (MIP-101 and Clone A) were prelabelled with $^{125}$I-IdUdR and injected intrasplenically into nude mice that had received a systemic injection of either 40 μg CEA or HBSS

**Table II** Enhancement of the experimental hepatic metastasis of human colorectal carcinoma cell lines by CEA

| | | | *No pretreatment* | | *HBSS* | | *CEA* | | |
|---|---|---|---|---|---|---|---|---|---|
| *Tumour* | *CEA[a]* | *DOS[b]* | *Mice w/ liver Mets[c]* | *Total tumour burden[d]* | *Mice w/ liver mets* | *Total tumour burden* | *Mice w/ liver mets* | *Total tumour burden* | *P<* |
| Clone A | 0 | 60 | 1/12 (8) | 0 × 11,II | 0/18 (0) | 0 × 18 | 6/19 (32) | 0 × 11,0* × 2, II × 4,II*,III* | 0.001 |
| MIP-101 | 0 | 65 | 1/20 (5) | 0 × 19,II | 0/16 (0) | 0 × 16 | 2/16 (13) | 0 × 9 0* × 5,I,II* | NS |
| CCL 235 | 65 | 80 | 5/10 (50) | 0 × 4,0*, II × 4,II* | 8/9 (88) | 0,II × 7, II* | 5/8 (63) | 0,0,0*, II × 4,II* | NS |
| CCL 188 | 97 | 33 | 4/11 (36) | 0 × 7, II × 4 | 4/10 (40) | 0 × 6,II × 2, III,III* | 4/40 (40) | 0 × 5,II, III × 2,IV | NS |
| HT-29 | 300 | 50 | 9/10 (90) | 0,II × 9 | 10/10 (100) | II × 9,III | 8/10 (80) | 0 × 2, II × 7,III | NS |

[a]CEA production is measured by enzyme immunoassay of the spent medium and expressed as ng CEA/$10^6$ cells/day. [b]DOS (day of sacrifice) is the day that mice were sacrificed for autopsy and is less than 90 if mice within a group became moribund. [c]The number of mice with one or more metastases in the liver divided by the total number of mice in the experiments (mice w/mets) and expressed as the percentage of mice with metastases (% mets). [d]Tumour burden in the liver is based on the grading system of Giavazzi *et al.* (22). [e]Groups of nude mice were injected in the dorsal tail vein with 40 μg of CEA in 0.2 ml of HBSS (CEA) or 0.2 ml of HBSS alone (HBSS) or not pretreated (None) and 30 min later mice received viable tumour cells injected intrasplenically. Mice were sacrificed 90 days later unless they became moribund. Experimental metastases were confirmed by histology. Experimental metastases were noted in the lungs of those mice marked with an asterisk. *P* values determined by Chi square on CEA *vs* the None control.

**Table IV** The effect of CEA on natural host effector cells in an *in vitro* cytotoxicity assay

| | *% release at 4 h* | | | | *% release at 20 h* | | | |
|---|---|---|---|---|---|---|---|---|
| | *Control* | *CEA* ($\mu$g ml$^{-1}$)$^{b}$ | | | *Control* | *CEA* 9$\mu$g ml$^{-1}$)$^{b}$ | | |
| *Tumour* | *Release*$^{a}$ | *0* | *0.5* | *1.0* | *Release*$^{a}$ | *0* | *0.5* | *1.0* |
| YAC-1 | 6.1 ± 0.5 | **10.4 ± 0.4** | **10.5 ± 0.4** | **10.1 ± 0.3** | 16.8 ± 0.6 | **29.2 ± 1.0** | **29.4 ± 1.7** | **33.6 ± 1.1** |
| KM-12c | 4.2 ± 0.1 | 4.0 ± 0.2 | 4.2 ± 0.2 | 4.1 ± 0.1 | 9.0 ± 0.2 | 8.9 ± 0.4 | 9.4 ± 0.3 | 9.5 ± 0.3 |
| MIP-101 | 6.5 ± 0.3 | 6.7 ± 0.3 | 6.8 ± 0.2 | 6.9 ± 0.2 | 21.4 ± 0.8 | 21.7 ± 0.8 | 21.8 ± 0.5 | 22.0 ± 0.6 |

[a]Control release is the spontaneous release and is the per cent of $^{51}$Cr released by labelled tumour cells in the absence of spleen cells. [b]Spleen cells were harvested from BALB/c mice and cocultured for 4 or 20 h with tumour cells that had been prelabelled with $^{51}$Cr at an effector:target ratio of 40:1 at 37°C with the indicated amount of CEA in the incubation medium. Assays were performed in quadruplicate. Results are expressed as Mean ± s.e.m. of the percentage of label released by tumour cells. Means ± s.e.m. in bold print are significantly different from the spontaneous release control by ANOVA and Scheffe F test at $P<0.001$.

**Table V** Retention of human colorectal carcinoma cells in the livers and other organs of nude mice pretreated with CEA intravenously

| | *% Injected tumour cells in each organ at 4 h* | | | | | |
|---|---|---|---|---|---|---|
| | *Liver* | | *Lung* | | *Spleen* | |
| *Tumour* | *CEA* | *HBSS* | *CEA* | *HBSS* | *CEA* | *HBSS* |
| MIP-101 | **61 ± 2** | 27 ± 7$^{a}$ | **0.9 ± 0.2** | 0.4 ± 0.1$^{b}$ | 6 ± 1 | 6 ± 2 |
| Clone A | **67 ± 7** | 29 ± 8$^{a}$ | 0.4 ± 0.1 | 0.3 ± 0.1 | 4 ± 1 | 2 ± 1 |
| CCL 188 | 43 ± 5 | 47 ± 5 | 0.3 ± 0.1 | 0.4 ± 0.1 | 10 ± 2 | 10 ± 1 |

$2 \times 10^{6}$ carcinoma cells that had been prelabelled with $^{125}$I-IdUdR were injected intrasplenically into groups of three athymic nude mice 30 min after the intravenous injection of either 40 $\mu$g of CEA in 0.2 ml of HBSS or 0.20 ml of HBSS alone, as described in Materials and methods. Results are Mean ± s.e.m. of the per cent of injected cells in organs at 4 h. $^{a}P<0.01$ and $^{b}P<0.05$ between CEA pretreated and HBSS Controls as determined by two-tailed upaired Student *t* test.

30 min earlier. The mice were sacrificed 4 h later and the livers, lungs, and other organs counted for radioactivity. The livers of mice that received HBSS contained more CCL 188 cells than Clone A or MIP-101 cells 4 h after injection (Table V). In contrast, CEA pretreatment significantly increased the number of MIP-101 and Clone A cells in liver but did not effect the retention of CCL 188 cells (Table V). In fact, there were more MIP-101 or Clone A cells in the livers of CEA-pretreated mice than in either group of mice that received CCL 188 cells. There was no significant difference in the distribution of tumour cells from any of the lines of the kidneys, spleen, or intestine (Table V and data not shown). There was also a significant doubling in the per cent of MIP-101 cells in the lungs of CEA pretreated mice compared to control mice, although the retention of Clone A and CCL 188 cells in the lungs was not effected by CEA pretreatment (Table V).

The effect of CEA pretreatment on tumour cell retention in the liver may be a nonspecific mechanical effect that retains tumour cells in the liver after they initially arrest in the hepatic sinusoid. To test this we injected $^{125}$I-albumin-labelled 2.8 $\mu$ microspheres intrasplenically into mice that were

**Figure 2** Effect of CEA pretreatment on the retention of micro-beads in the liver and other organs. $^{125}$I-labelled 2.8 $\mu$ beads were injected into groups of 3–10 athymic nude mice that had received 40 $\mu$g of CEA in 0.2 ml of HBSS or HBSS alone. Mice were sacrificed at different times and their organs collected and radioactivity counted. Values represent means ± s.e.m. of the per cent of injected dose of beads in the organ at the time of sacrifice. (□), (■), and (■) the percentage of beads in the livers, lungs, and kidneys, respectively, of HBSS pretreated mice and (◆), (◇), and (□) the percentage of beads in the livers, lungs, and kidneys, respectively, of CEA pretreated mice. The amount of radioactivity in the kidneys is similar to the amount of 0.3 ml of blood and in the gastrointestinal tract.

**Figure 3** Relationship between retention of colorectal carcinoma cells within the livers of nude mice and the incidence of experimental metastasis. $^{125}$I-IdUdR prelabelled carcinoma cells were injected intrasplenically into groups of three nude mice that were either pretreated with CEA or HBSS and the percentage of tumour cells present in the liver 4 h later calculated as described in 'Materials and methods'. This percentage was then plotted against the per cent of mice that developed experimental metastases for each cell line that received either CEA or HBSS. The cell lines are moderately (CCL-188), weakly (KM-12c, MIP-101, Clone A) or nonmetastatic (HC 2998), and all (□) are included in the regression equation whose characteristics are: % Mets = 0.899 × % Cells − 11.2, R = 0.89, $P<0.001$. Data for % Cells in liver for KM-12c and HC 2998 are in ref. (Hostetter *et al.*, 1990*b*) while the incidence of metastases are from Table III.

pretreated with either 40 μg of CEA or HBSS and then examined their distribution in the liver and other organs up to 6 h later. There was no difference in the number of microspheres in the liver up to 6 h after intrasplenic injection (Figure 2). There was a small percentage of radioactivity that accumulated in the lungs, blood, and other organs that may represent particles that are released from the liver after an initial trapping in the liver (Figure 2). Clearly, CEA pretreatment does not alter the mechanical trapping of inert beads in the liver. Thus, the ability of CEA pretreatment to double the number of weakly metastatic tumour cells persisting in the liver 4 h after intrasplenic injection is the result of a more specific biological interaction among the tumour cells, hepatic cells, and CEA.

The importance of the number of cells in the liver a few hours after entry into the liver is underscored by comparing the percentage of cells in the liver at 4 h to the incidence of experimental liver and lung metastases. Indeed, when the results of our earlier studies (Hostetter *et al.*, 1990*b*) are included, there is a linear relationship between the formation of experimental metastases and the percentage of tumour cells in the liver 4 h after intrasplenic injection (Figure 3). The effect of CEA upon the retention of tumour cells is lost within 24 h because there is no significant difference between the number of KM-12c cells in the liver of CEA-pretreated mice compared to HBSS-pretreated mice (data not shown). Thus, the effect of CEA upon the production of experimental metastases by weakly metastatic carcinoma cells is associated with the early persistence of colorectal carcinoma cells within the liver.

### Discussion

The present study extends our earlier observations that CEA injected intravenously into nude mice promotes the formation of experimental metastases by human colorectal carcinomas in nude mice (Hostetter *et al.*, 1990*a*,*b*) and gives insight into possible mechanisms. The distribution of CEA in athymic nude mouse liver after intravenous injection is similar to that previously reported in immunocompetent mouse liver by Toth *et al.* (1982). Interestingly, although murine Kupffer cells and hepatocytes contained CEA on indirect immunoperoxidase staining of the liver within 30–60 min of the intravenous CEA injection, the CEA was retricted to Kupffer cells at 4 h when the liver contained only 75 ng of CEA per mg cell protein. Kupffer cells contain unique endocytic compartments in which material that is poorly degraded is stored for extended periods (Kindberg *et al.*, 1991). This may account for the presence of CEA in Kupffer cells at 4 h and possibly longer. CEA in human liver adjacent to colorectal carcinoma metastases was also regionally distributed in the intralobular or midzonal areas, although not as symmetrically as in the nude mouse liver. Concentrations greater than 10 ng ml$^{-1}$ of CEA have been observed in the bile that drains the livers of patients with primary colon cancers who do not have clinical evidence of metastasis (Yeatman *et al.*, 1989). This finding combined with our observation in a patient with metastases and an elevated serum CEA suggests that the concentration of CEA achieved in the liver is comparable to that used *in vitro* for the adhesion of cells to CEA attached to a solid phase (Hostetter *et al.*, 1990*b*).

Our data demonstrate (1) that the propensity to form metastases is dependent in part on the number of cells that persist in the liver several hours after initial arrest and (2) that CEA pretreatment increases the number of weakly metastatic tumour cells present in the liver 4 h after intrasplenic injection. Fidler has shown that the technique of using cells labelled endogenously with $^{125}$IdUdr identifies intact cells within an organ (Fidler, 1970). Morikawa *et al.* (1988*b*) and Hostetter *et al.* (1990*b*) demonstrated that the percentage of cells in an organ 4 or more hours after systemic injection is associated with the ability of those tumour cells to produce metastases. Only 5% of the nonmetastatic HC 2998 human colon cancer cells were present in the liver at 4 h which was much less than the approximately 20% of weakly metastatic KM-12c cells and over 40% of highly metastatic mHC 1410 cells present in liver at that time (Hostetter *et al.*, 1990). Since the HC 2998 cells are not present in blood or lungs and had the same percentage of cells in the spleen as the metastatic cell lines at 4 h (data not shown), it is likely that the nonmetastatic HC 2998 tumour cells are lysed during the first few hours in the liver.

On entering the liver, tumour cells will be trapped in the sinusoids (Weiss, 1990). The vast majority of these cells are rapidly eliminated through various specific and nonspecific defense mechanisms with the result that very few cells are left to establish metastases. Nonetheless, some tumour cells survive in the hepatic microcirculation, leave the liver, and successfully establish metastases elsewhere. For example, both MIP-101 cells (Wagner *et al.*, 1990) and Clone A cells (Table II) produced lung metastases in CEA pretreated mice that lacked liver metastases on microscopic examination. Even though the process of metastasis is inefficient (Weiss, 1990), it stands to reason that the more viable tumour cells that persist in the liver after an initial phase of trapping and elimination, then the more metastases may be formed later when these cells proliferate. This also suggests that CEA pretreatment enhances the ability of tumour cells to resist the various mechanisms of the host.

CEA may increase the persistence of weakly metastatic carcinoma cell lines through several different mechanisms. Hostetter *et al.* (1990*b*) have shown that KM-12c binds to CEA attached to a solid phase. Thus, adhesion to CEA attached to a surface may be an important mechanism. However, the other weakly metastatic cell lines that respond *in vivo* to CEA pretreatment (MIP-101 and Clone A) do not bind to CEA on a solid phase, although both the highly metastatic HT-29 and nonmetastatic HC-2998 do bind *in vitro* to CEA attached to a solid phase (Meterissian *et al.*, 1991). Microbeads coated with either albumin (Figure 2) or with CEA (data not shown) were not preferentially retained in the livers of mice that had received CEA intravenously. Thus, while adhesion to cell surface CEA is an attractive hypothesis and may be important for the promotion of metastasis by one colorectal carcinoma line (KM-12c), it does not explain the effects of CEA upon the two other weakly metastatic lines.

CEA affects other aspects of the host-tumour relationship apart from adhesion. We have shown previously that CEA inhibits plaque forming cell responses to sheep erythrocytes in mice and the production of antibodies to human tumour-associated antigens by athymic nude mice (Hostetter *et al.*, 1990*a*). CEA also induced human T cells to release immunosuppressive factors (Medoff *et al.*, 1984) and to directly inhibit lymphocyte proliferation (Hakim, 1984). It is possible that the metastatic potential of MIP-101 and Clone A may be enhanced by CEA-mediated inhibition of the immune responses to nude mice. Nude mice make T-independent responses that may support antibody-dependent cell-mediated cytotoxicity and prevent the growth of experimental metastases. CEA may inhibit the production of antibodies that are deleterious to the establishment of liver colonies by weakly metastatic cell lines. However, our data suggest that CEA does not alter the lytic function of NK cells by mouse spleen cells. It is doubtful that the cytostatic or cytotoxic activity of unstimulated Kupffer cells in normal nude mice will be effected by CEA pretreatment. Thus, it seems unlikely that inhibition of natural host resistance mechanisms participate in CEA-mediated enhancement of experimental metastasis.

In summary, CEA injected intravenously into nude mice facilitates the growth of tumour in liver and lung by three of eight colorectal carcinoma lines. The cell lines enhanced by CEA are all weakly metastatic and CEA increases the persistence of these tumour cells in the liver. Since Kupffer cells in rats and humans have both 35 and 80 kDa receptors for CEA (Thomas *et al.*, 1991), CEA-expressing tumour cells may bind to Kupffer cells using such a CEA:Kupffer cell receptor interaction. CEA secreted by the primary tumour may also enhance the metastasis of weakly metastatic cell lines by occupying Kupffer cell receptors and binding with CEA on tumour cells. While CEA-mediated adhesion is an attractive hypothesis to explain the effect of CEA upon metastasis, two of the CEA-enhancable cell lines (MIP-101 and Clone A) do not bind to purified CEA attached to a solid phase (Meterissian et al., 1991). It is possible, therefore, that some other adhesion molecule or molecules may be involved in the persistence of tumour cells within liver, possibly through activation of Kupffer cells that metabolise CEA. This activation may lead to cytokine release that enhances expression of another adhesion receptor or ligand to which the carcinoma cells may bind. Alternatively, CEA may have some other, as yet undefined, action that promotes the survival of tumour cells within the liver. Whatever mechanism is operative, CEA is a product of tumour cells that can facilitate the production of metastases within a subset of colorectal carcinoma cell lines.

Supported by grants CA42587 (JMJ), CA44583 (PT), and CA44704 (GS Jr) from the U.S. Department of Health and Human Services and NAG9-972 from the National Aeronautics and Space Administration (JMJ).

### References

BENCHIMOL, S., FUKS, A., JOTHY, S., BEAUCHEMIN, N., SHIROTA, K. & STANNERS, C.P. (1989). Carcinoembryonic antigen, a human tumour marker, functions as an intercellular adhesion molecule. *Cell*, **57**, 327.

BERLING, B., KOLBINGER, F., GRUNERT, F., THOMPSON, J.A., BROMBACHER, F., BUCHEGGER, F., VON KLEIST, S. & ZIMMERMANN, W. (1990). Cloning of a carcinoembryonic antigen gene family member expressed in leukocytes of chronic myeloid leukeamia patients and bone marrow. *Cancer Res.*, **50**, 6534.

BESSELL, E.M., THOMAS, P. & WESTWOOD, J.H. (1975). Multiple Smith degradations of carcinoembryonic antigen (CEA) and asialo CEA. *Carbohydrate Res.*, **45**, 257.

BYRN, R., MEDREK, P., THOMAS, P., JEANLOZ, R. & ZAMCHECK, N. (1985). Effect of heterogeneity of CEA on liver cell membrane binding and its kinetics of removal from the circulation. *Cancer Res.*, **45**, 3137.

DEXTER, D.L., BARBOSA, J.A. & CALABRESI, P. (1979). N,N-dimethylformamide-induced alteration of cell culture characteristics and loss of tumorigenicity in cultured human colon carcinoma cells. *Cancer Res.*, **39**, 1020.

FIDLER, I.J. (1970). Metastasis: quantitative analysis of distribution and fate and tumor emboli labelled with $^{125}$I-5-Iodo-2'-deoxyuridine. *J. Natl Cancer Inst.*, **45**, 773.

GIAVAZZI, R., CAMPBELL, D.E., JESSUP, J.M., CLEARY, K. & FIDLER, I.J. (1986). Metastatic behavior of tumor cells isolated from primary and metastatic human colorectal carcinomas implanted into different sites in nude mice. *Cancer Res.*, **46**, 1928.

GOSLIN, R., STEEL, G. Jr, MACINTYRE, J., MAYER, R., SUGARBAKER, P., CLEGHORN, K., WILSON, R. & ZAMCHECK, N. (1980). The use of preoperative plasma CEA levels for the stratification of patients after curative resection of colorectal cancers. *Ann. Surg.*, **192**, 747.

HAKIM, A. (1984). CEA antigen, a tumor associated glycoprotein induces defective lymphocyte function. *Neoplasm*, **31**, 385.

HOSTETTER, R.B., CAMPBELL, D.E., CHI, K.-F., KERCKHOFF, S., CLEARY, K.R., ULLRICH, S., THOMAS, P. & JESSUP, J.M. (1990*a*). Carcinoembryonic antigen enhances metastatic potential of human colorectal carcinoma. *Arch. Surg.*, **125**, 300.

HOSTETTER, R.B., AUGUSTUS, L.B., MANKARIOUS, R., CHI, K.F., FAN, D., TOTH, C., THOMAS, P. & JESSUP, J.M. (1990*b*). Carcinoembryonic antigen as a selective enhancer of colorectal cancer metastases. *J. Natl Cancer Inst.*, **82**, 380.

JESSUP, J.M., MACEK, C.M., KAHAN, B.D. & PELLIS, N.R. (1981). Effect of murine tumors upon delayed hypersensitivity to dinitrochlorobenzene. III. *In vivo* activity of the nonspecific suppressor cell. J. Immunol., **127**, 2183.

KINDBERG, G.M., TOLLESHAUG, H., GJOEN, T. & BERG, T. (1991). Lysosomal and endosomal heterogeneity in the liver: a comparison of the intracellular pathway of endocytosis in rat liver cells. *Hepatology*, **13**, 254.

MEDOFF, J.R., JEGASOTHY, B.V. & ROCHE, J.K. (1984). Carcinoembryonic antigen-induced release of a suppressor factor from normal human lymphocytes *in vitro*. *Cancer Res.*, **44**, 5822.

METERISSIAN, S., FORD, R., TOTH, C.A., THOMAS, P., STEELE, G. & JESSUP, J.M. (1991). Carcinoembryonic antigen is an adhesion molecule for colorectal carcinoma. *Proc. Am. Assoc. Cancer Res.*, **32**, 72.

MORIKAWA, K., WALKER, S.M., JESSUP, J.M. & FIDLER, I.J. (1988*a*). *In vivo* selection of highly metastatic cells from surgical specimens of different human colon carcinomas implanted into nude mice. *Cancer Res.*, **48**, 1943.

MORIKAWA, K., WALKER, S.M., NAKAJIMA, M., PATHAK, S., JESSUP, J.M. & FIDLER, I.J. (1988*b*). Influence of organ environment on the growth, selection, and metastasis of human colon carcinoma cells in nude mice. *Cancer Res.*, **48**, 6863.

MURRAY, D., HRENO, A., DUTTON, J. & HAMPSON, L.G. (1975). Prognosis in colon cancer: a pathologic reassessment. *Arch. Surg.*, **110**, 908.

NILES, R.M., WILHELM, S.A., STEELE, G.D. Jr, THOMAS, P. & ZAMCHECK, N. (1987). Isolation and characterization of an undifferentiated human colon carcinoma cell line (MIP-101). *Cancer Invest.*, **5**, 545.

OIKAWA, S., INUZUKA, C., KUROKI, M., MATSUOKA, Y., KOSAKI, G. & NAKAZATO, H. (1989). Cell adhesion activity of non-specific cross-reacting antigen (NCA) and carcinoembryonic antigen (CEA) expressed on CHO cell surface: homophilic and heterophilic adhesion. *Biochem. Biophys. Res. Commun.*, **164**, 39.

SPRATT, J.S. & SPJUT, H.J. (1967). Prevalence and prognosis of individual clinical and pathological variables associated with colorectal carcinoma. *Cancer*, **20**, 1976.

THOMAS, P. & SUMMERS, J.W. (1978). The biliary excretion of circulating asialoglycoproteins in the rat. *Biochem. Biophys. Res. Commun.*, **80**, 335.

THOMAS, P., TOTH, C.A., FOX, E.S. & STEELE, G. (1991). Carcinoembryonic antigen binding proteins from Kupffer cells. In *Cells of the Hepatic Sinusoid.* Vol 3. The Krupffer Cell Foundation. Rijswijk. The Netherlands. **3**, 506.

TOTH, C.A., RAPOZA, A., ZAMCHECK, N., STEELE, G. & THOMAS, P. (1989*a*). Receptor-mediated endocytosis of carcinoembryonic antigen by rat alveolar macrophages *in vitro*. *J. Leukocyte Biol.*, **45**, 370.

TOTH, C.A., RAPOZA, A., KOWAL, A., STEELE, G. & THOMAS, P. (1989). Receptor mediated endocytosis by human Kupffer cells. *Biochem. Soc. Trans.*, **16**, 1027.

TOTH, C.A., THOMAS. P., BROITMAN, S.A. & ZAMCHECK, N. (1982). A new Kupffer cell receptor mediating plasma clearance of carcinoembryonic antigen by the rat. *Biochem. J.*, **204**, 377.

TOTH, C.A., THOMAS, P., BROITMAN, S.A. & ZAMCHECK, N. (1985). Receptor-mediated endocytosis of carcinoembryonic antigen by rat liver Kupffer cells. *Cancer Res.*, **45**, 392.

WAGNER, H.E., THOMAS, P., WOLF, B.C., ZAMCHECK, N., JESSUP, J.M. & STEELE, G.D. Jr (1990). Characterization of the tumorigenic and metastatic potential of a poorly differentiated human colon cancer cell line. *Invasion & Metastasis*, **10**, 253.

WANEBO, H.J., RAO, B., PINSKY, C.M., HOFFMAN, R.G., STEARNS, M., SCHWARTZ, M.K. & OETTGEN, H.F. (1978). Preoperative carcinoembryonic antigen level as a prognostic indicator in colorectal cancer. *N. Engl. J. Med.*, **299**, 448.

WEISS, L. (1990). Metastatic inefficiency. *Adv. Cancer Res.*, **54**, 159.

WILLIAMS, A.F. & BARCLAY, A.N. (1988). The immunoglobulin superfamily: domains for cell surface recognition. *Ann. Rev. Immunol.*, **6**, 381.

YEATMAN, T.J., KIMURA, A.K., COPELAND, E.M. & BLAND, K.I. (1989). Relationship between colorectal liver metastases and CEA levels in gallbladder bile. *Ann. Surg.*, **210**, 505.

ZHOU, H., FUKS, A. & STANNERS, C.P. (1990). Specificity of intracellular adhesion mediated by various members of the immunoglobulin supergene family. *Cell Growth & Differ.*, **1**, 209.